# Amalgam in England

**Published:** 1862-04

**Authors:** 


### AMALGAM IN ENGLAND.

We have not recently seen any thing more humiliating than a paper read (by Mr. Tomes, perhaps,) at a meeting of the Odontological Society, "*On certain conditions presented by Amalgams used in Filling Faulty Teeth.*"

It is not our purpose to give an extended notice of the paper. It is, in some respects, worthy of careful attention, and it is well written, as we would, of course, expect. But just think of this statement:—"I, in common, I believe, with many others, purchase and use materials prepared by Messrs. Ash or those of other makers, without knowing the proportion the incorporated metals bear to each other."

Now, to think of the leading dentists of England going *blindly* into such quackery! To use amalgams at all is almost unpardonable; but to use them without a knowledge of their composition—we regret that we heard of it. W.

